# Dietary supplementation by older adults in southern China: a hospital outpatient clinic study

**DOI:** 10.1186/1472-6882-9-39

**Published:** 2009-10-20

**Authors:** Wenbin Liang, Andy H Lee, Colin W Binns

**Affiliations:** 1 School of Public Health, Curtin Health Innovation Research Institute, Curtin University of Technology, Perth, WA, Australia; 2 National Drug Research Institute, Curtin University of Technology, Perth, WA, Australia

## Abstract

**Background:**

There has been little knowledge about dietary supplementation by the Chinese elderly. The aim of this cross-sectional study was to investigate the usage of dietary supplements by older adults in southern China.

**Methods:**

A total of 600 community-dwelling older adults were recruited from the outpatient clinics of three major hospitals in Foshan city between July 2007 and July 2008. Face-to-face interviews of participants were conducted to obtain information on demographics, lifestyle and dietary supplements use. Frequency and duration of usage were recorded for six categories of dietary supplements.

**Results:**

Among the 446 consented participants (241 men and 205 women) who were over 55 years of age, 19.1% consumed one or more types of dietary supplements. The prevalence of usage was significantly higher (p = 0.008) for females (24.4%) than for males (14.5%). Dietary supplements were more likely to be consumed by non-smokers (p = 0.021) and those with hyperlipidemia (p = 0.003). The most popular supplement among users was calcium (53%). The majority (71%) of the users consumed supplements on a regular basis at one or more times per day, with an average duration of 2.95 (SD 4.80) years.

**Conclusion:**

The overall prevalence of dietary supplementation in this older Chinese population was considerably lower than those in other Asia-Pacific countries.

## Background

Dietary supplements, especially multivitamins and mineral supplements, have been widely consumed in many developed countries even though their effectiveness is unclear [1-3]. A randomized controlled trial suggested that regular intake of multivitamin and multi-mineral supplements may reduce infection among patients with diabetes [4]. The protective effect of multivitamin supplements intake against the progression of HIV was also observed in a randomized controlled trial [5]. However, a meta-analysis of five randomized control trials found no significant beneficial effect of multivitamin and multi-mineral supplements [6]. In another study that investigated the pooled data from eight prospective studies, no association was evident between the lung cancer risk and the use of folate, vitamins A, C, E, and multivitamins [7]. On the contrary, a meta-analysis of 19 trials concluded that high-dosage of vitamin E supplements could actually lead to an increased mortality [8]. In spite of these and other contradictory evidence, use of dietary supplements remains popular in Western populations.

The Chinese economy has been growing rapidly over the past 20 years [9]. Various types of dietary supplements are now readily available in the Chinese market. It is unclear whether the use of dietary supplements affects the health of the Chinese adults. There have been little details documenting their usage of dietary supplements in the literature. Therefore, the objective of the present study was to ascertain the prevalence and types of dietary supplements used by older adults in southern China. The focus was on the usage of tablet and capsule forms of dietary supplements, whereas traditional Chinese medicines, herbals and botanicals were not investigated.

The findings would be important for monitoring the consumption trends in relation to disease prevention and in comparison with other Asia-Pacific countries.

## Methods

### Study design and participants

A cross-sectional study was conducted between July 2007 and July 2008 in Foshan city located in the Guangdong Province of southern China. A total of 600 community-dwelling older adults were initially recruited from the outpatient clinics of three major hospitals, namely, the First People's Hospital of Shunde, First People's Hospital of Nanhai, and Second People's Hospital of Foshan. These three hospitals were similar in size and types of medical treatments provided. Subjects from the clinics of the Departments of Gastroenterology, Dermatology, Chinese Medicine, Urology and Otolaryngology were approached at random while they were waiting for medical service. To be eligible, subjects must be 55 years of age or over and have resided in Foshan for at least the past five years. Those subjects with a diagnosis of Alzheimer's disease or had been on long-term modification of diet for medical reasons were excluded. Long term dietary modification for medical reasons refers to people who stopped eating certain foods for reasons that include allergy, chemotherapy treatment of cancer, and surgery of the digestive system. Of the 512 subjects who signed the consent form and agreed to take part, 446 participants (241 men and 205 women) completed the interview and were available for analysis, representing a final response rate of 74.3%. No significant differences in mean age and gender distributions were found between participants and those who declined or subsequently withdrew from the study.

### Interview and questionnaire

The study protocol was approved by the participating hospitals and the Human Research Ethics Committee of the researchers' institution. All participants were assured confidentiality and their right to withdraw without prejudice before obtaining their formal consent. Face-to-face interviews of participants were conducted by the first author at the outpatient clinics.

A structured questionnaire was administered to obtain demographic and lifestyle characteristics including age, gender, weight (kg), height (m), education level (primary school; secondary school or above), occupation before retirement (professional; others), smoking status (non-smoker; smoker), and alcohol drinking status (non-drinker; drinker). Self-reported height and weight measurements and health conditions such as the presence of hypertension, hyperlipidemia and diabetes were confirmed with medical records whenever available; see Additional File 1.

Participants were asked whether they had used dietary supplements over the last year. Information on the frequency and duration of usage was then recorded for six categories of common dietary supplements, namely, multivitamins and minerals, vitamin C, vitamin E, fish oil, calcium, and miscellaneous (such as vitamin A, garlic, ganoderma lucidum, propolis, cordyceps, ginseng and panax notoginseng).

### Statistical analysis

Descriptive statistics and chi-square tests were performed to summarise and compare characteristics between dietary supplement users and non-users. The prevalence of dietary supplementation by gender was tabulated, as well as the frequency and duration of usage for each category of supplements among users. Logistic regression analysis was conducted to examine whether the reported chronic conditions would affect the usage of dietary supplements. All statistical analyses were undertaken using the SPSS for Windows package version 13.

## Results

Sample demographic and lifestyle characteristics by user status are presented in Table 1. The average age of the participants was 69.5 (SD 7.4) years with over half (53%) of them between 64-74 years of age. The overall prevalence of dietary supplement use was 19.1%, but significant difference in prevalence was found between male (14.5%) and female (24.4%) participants (p = 0.008). Dietary supplements were more likely to be consumed by non-smokers (p = 0.021) as well as those who had been diagnosed with hyperlipidemia (p = 0.003). Logistic regression analysis further confirmed that the presence of hyperlipidemia was significantly associated with dietary supplement usage (p = 0.023), with adjusted odds ratio 2.09 (95% confidence interval 1.11 to 3.94) after accounting for the available demographic and lifestyle factors. However, no association was found between dietary supplementation and age, body mass index, education level, occupation, alcohol drinking, and presence of hypertension and diabetes.

**Table 1:** Characteristics of participants by dietary supplementation status

**Characteristic**	**Dietary supplementation**	
	**Non-user (n = 361)**	**User (n = 85)**
Gender *		
Male	206 (57.1%)	35 (41.2%)
Female	155 (42.9%)	50 (58.8%)
Age (years)		
55-64	85 (23.6%)	22 (25.9%)
65-74	192 (53.2%)	46 (54.1%)
74-84	67 (18.6%)	15 (17.7%)
≥ 85	17 (4.7%)	2 (2.4%)
Body mass index (kg/m^2^)		
< 24	218 (60.4%)	46 (54.1%)
≥ 24	143 (39.6%)	39 (45.9%)
Educational level		
Primary school	155 (42.9%)	31 (36.5%)
Secondary school or above	206 (57.1%)	54 (63.5%)
Occupation before retirement		
Professional	136 (37.7%)	39 (45.9%)
Others	225 (62.3%)	46 (54.1%)
Smoking status *		
Smoker	78 (21.6%)	9 (10.6%)
Non-smoker	283 (78.4%)	76 (89.4%)
Alcohol drinking status		
Drinker	150 (41.6%)	28 (32.9%)
Non-drinker	211 (58.5%)	57 (67.1%)
Presence of hyperlipidemia *		
Yes	38 (10.5%)	19 (22.4%)
No	323 (89.5%)	66 (77.7%)
Presence of hypertension		
Yes	94 (26.0%)	31 (36.5%)
No	267 (74.0%)	54 (63.5%)
Presence of diabetes		
Yes	10 (2.8%)	2 (2.4%)
No	351 (97.2%)	83 (97.7%)

* Significant chi-square test of association between characteristic and dietary supplementation status

Table 2 shows that the most popular supplement among users was calcium (53%), especially for females (62%), followed by multivitamins and minerals (32%). The majority (71%) of the users consumed supplements on a regular basis at one or more times per day, with an average duration of 2.95 (SD 4.80) years. However, as shown in Table 2, the average duration of consumption varied considerably from 2 to 7.5 years among the different categories of supplements.

**Table 2:** Prevalence, frequency and duration of dietary supplements intake by users (n = 85)

**Category**	**Male users** **(n = 35)**	**Female users** **(n = 50)**	**Both genders** **(n = 85)**		
			**Users**	**Prevalence of daily use**	**Mean duration (SD) in months**
Multivitamins and minerals	11 (31.4%)	16 (32%)	27 (31.8%)	70.4%	66.1 (100.2)
Vitamin C	5 (14.3%)	7 (14%)	12 (14.1%)	91.7%	89.4 (135.6)
Vitamin E	4 (11.4%)	9 (18%)	13 (15.3%)	76.9%	42.5 (52.4)
Fish oil	9 (25.7%)	16 (32%)	25 (29.4%)	66.7%	28.2 (36.1)
Calcium	14 (40.0%)	31 (62%)	45 (52.9%)	66.7%	24.3 (23.2)
Miscellaneous*	6 (17.1%)	6 (12%)	12 (14.1%)	75.0%	29.9 (50.8)

* includes vitamin A, garlic, ganoderma lucidum, propolis, cordyceps, ginseng and panax notoginseng

## Discussion

This study provides the first report of dietary supplementation in the southern Chinese population. The estimated prevalence of 19.1% was considerably lower than those of other countries in the Asia-Pacific region [10-16]; see Table 3. In particular, 34.9% of Taiwanese females and 30.1% of Taiwanese males over the age of 65 consumed dietary supplements [10]. Nevertheless, the observed higher prevalence of dietary supplementation by southern Chinese elderly females was consistent with other countries. Taiwanese consumed calcium, vitamin E, vitamin C, vitamin B-complex, fish oil, omega-3 fatty acids, chicken extracts, with ginseng being the only traditional Chinese medicine reported [10]. Our recent study in Japan investigated five categories of dietary supplements, namely, multivitamins, beta-carotene, vitamin C, vitamin E and miscellaneous, and found vinegar and energy drink the most popular within the miscellaneous category [11]. Another study in Australia considered vitamins B, E, C plus bioflavonoid, multivitamins, multi-minerals, fish oil, garlic, calcium, zinc and Gingko biloba [12]. The National Nutrition Survey in New Zealand, conducted in 1997, included multivitamins, multi-minerals, vitamins A, B, C, D and E, beta-carotene and single minerals namely iron, calcium, magnesium, potassium, selenium, and zinc [13]. A Korean study investigated five categories of supplements: foods for special dietary use, health foods, herb and Chinese medicine, vitamin and mineral supplements, dietary supplements [15]. However, the studies in Indonesia [14] and Malaysia [16] did not specify clearly the types of dietary supplements being used.

**Table 3:** Prevalence of dietary supplementation by older adults in the Asia-Pacific region

**Country**	**Year**	**Sample size**	**Age range**	**Both genders**	**Male**	**Female**
Southern China	2008	446	55	19.1%	14.5%	24.4%
Taiwan [10]	2000	1937	65+	32.5%	30.1%	34.9%
Japan [11]	2006	572	55-75	45.8%	41.7%	52.5%
Australia [12]	2001	1263	65+	43%	35%	52%
New Zealand [13]	1997	814	65+	36.1%	24.7%	43.7%
Indonesia [14]	1996	204	60-75	25.0%	24.7%	25.2%
Korea [15]	2000	2188	50+	30.2%	28.5%	30.7%
Malaysia [16]	2005	60	50+	83.1%	NA	NA

Calcium was found to be the most popular supplement among our study participants. Dietary calcium intake by Chinese has been estimated to be lower than Western populations [17] at 430 mg/day, while the average consumption of milk is only 4 g/day [18]. The low consumption of dairy products is partly due to their intolerance to lactose [19]. The presence of hyperlipidemia was found to be associated with dietary supplement usage. The literature has suggested that insufficient calcium intake can increase the risk of cardiovascular diseases [20,21]. Intake of calcium supplements could also improve bone health and reduce the risk of osteoporosis for Chinese elderly [19].

In this study, information on dietary supplementation was obtained from self report. As with other surveys of elderly subjects, the responses from our participants inevitably incurred some recall error. Therefore, face-to-face interviews were used to increase the response rate and to improve the accuracy of their answers. All interviews were conducted by the first author to eliminate inter-interviewer bias.

A limitation of our study was the lack of qualitative data on the perception and belief behind dietary supplement use due to time constraints. We recommend this to be the topic of a further study. Another limitation concerned the selection bias of our convenience sample. Subjects were approached at random while waiting for medical service. We recruited these voluntary participants from hospital outpatient departments rather than community health clinics because the majority of urban Chinese people seek medical service from major hospitals [22]. Currently, there is no medical referral system in China and the quality of service provided by community clinics is low [23]. It is not uncommon for a person to seek medical advice from a hospital specialist, such as a cardiologist for influenza or other minor symptoms. Consequently, the place of interview for each individual was not recorded. Moreover, community-based randomised sampling would be difficult to implement with high refusal rate expected in practice.

## Conclusion

The prevalence of dietary supplements usage by older Chinese adults was considerably lower than those in other Asia-Pacific countries. Nevertheless, almost 20% took supplements within the past year and 71% of users did so on a regular basis. This study provided useful baseline data for comparison with future surveys to monitor the consumption trends in southern China.

## Competing interests

The authors declare that they have no competing interests.

## Authors' contributions

WBL collected the data and drafted the manuscript. AHL analysed the data and revised the manuscript. CWB designed the study and revised the manuscript. All authors read and approved the final version of the paper.

## Pre-publication history

The pre-publication history for this paper can be accessed here:



## Supplementary Material

Additional file 1 **Questionnaire**. Simple questionnaire to obtain information on demographic characteristics and dietary supplement usage. Click here for file
