# Supplementary material for: Dietary supplementation by older adults in southern China: a hospital outpatient clinic study
Source: BMC Complement Altern Med. 2009 Oct 20;9:39. doi: 10.1186/1472-6882-9-39 (PMC2770031; doi:10.1186/1472-6882-9-39)
Supplement: Additional file 1 — Questionnaire. Simple questionnaire to obtain information on demographic characteristics and dietary supplement usage. [file 1472-6882-9-39-S1.DOC]

**Demographics**

1. Date of birth ______________ (day/month/year)

2. Gender 🞎 Male 🞎 Female

3. Weight ______________ kg

4. Height (no shoes) ______________ m

5. Education level 🞎 Primary school

🞎 Secondary school

🞎 College or university

6. Occupation before retirement

🞎 Professional (e.g. technician, clerical or administrative officer, manager, health workers, engineers)

🞎 Others (e.g. laborer, farmer, factory worker, unemployed, housewife, cook)

7. Smoking status 🞎 non-smoker 🞎 smoker

8. Average number of cigarettes smoked ______________ per day

9. Total number of years smoked ______________ years

10. Alcohol drinking status 🞎 non-drinker 🞎 drinker

# Medical history

11. Previous diagnosis of hypertension 🞎 no 🞎 yes

12. Previous diagnosis hyperlipidemia 🞎 no 🞎 yes

13. Previous diagnosis of diabetes 🞎 no 🞎 yes

**Dietary supplement use**

14. Take dietary supplements over the last year 🞎 no 🞎 yes

15. Dietary supplement usage by frequency and duration

| Supplement | ≤ 1 time /month | 2-3 times /month | 1 time /week | 2-3 times /week | 4-6 times /week | 1 time  /day | number of months |
| --- | --- | --- | --- | --- | --- | --- | --- |
| multivitamins & minerals |  |  |  |  |  |  |  |
| vitamin C |  |  |  |  |  |  |  |
| vitamin E |  |  |  |  |  |  |  |
| fish oil |  |  |  |  |  |  |  |
| calcium |  |  |  |  |  |  |  |
| miscellaneous |  |  |  |  |  |  |  |
| please specify: |  | | | | | | |
